# A proposed One Health approach to control yellow fever outbreaks in Uganda

**DOI:** 10.1186/s42522-024-00103-x

**Published:** 2024-05-23

**Authors:** Emmanuel Angmorteh Mensah, Samuel Ofori Gyasi, Fred Nsubuga, Walid Q. Alali

**Affiliations:** 1 Department of Biostatistics & Epidemiology, College of Public Health, East Tennessee State University, Johnson City, TN USA; 2 Department of Immunization, Vaccines and Biologicals, World Health Organization Country Office, Kampala, Uganda; 3 Division of Immunization and Vaccines, Ministry of Health, Kampala, Uganda

**Keywords:** YF, Uganda, One Health, Vector-borne disease, Africa

## Abstract

Yellow Fever (YF) is an acute viral hemorrhagic disease. Uganda is located within the Africa YF belt. Between 2019 and 2022, the Ugandan Health Authorities reported at least one outbreak of YF annually with an estimated 892 suspected cases, on average per year. The persistent recurrence of this disease raises significant concerns about the efficacy of current response strategies and prevention approaches. YF has been recognized as a One Health issue due to its interrelatedness with the animal and environmental domains. Monkeys have been recognized as the virus primary reservoir. The YF virus is transmitted through bites of infected *Aedes* or *Haemagogus* species mosquitoes between monkeys and humans. Human activities, monkey health, and environmental health issues (e.g., climate change and land use) impact YF incidence in Uganda. Additionally, disease control programs for other tropical diseases, such as mosquitoes control programs for malaria, impact YF incidence.

This review adopts the One Health approach to highlight the limitations in the existing segmented YF control and prevention strategies in Uganda, including the limited health sector surveillance, the geographically localized outbreak response efforts, the lack of a comprehensive vaccination program, the limited collaboration and communication among relevant national and international agencies, and the inadequate vector control practices. Through a One Health approach, we propose establishing a YF elimination taskforce. This taskforce would oversee coordination of YF elimination initiatives, including implementing a comprehensive surveillance system, conducting mass YF vaccination campaigns, integrating mosquito management strategies, and enhancing risk communication. It is anticipated that adopting the One Health approach will reduce the risk of YF incidence and outbreaks.

## Background

Yellow Fever (YF) is a mosquito-borne acute viral hemorrhagic infectious disease caused by an RNA virus belonging to the genus *Flavivirus* [1]^,^ [2]. The virus is transmitted to humans through the bite of infected *Aedes* mosquito species in Africa and *Hemagogus* mosquito species in South America [3]. As a vector-borne zoonotic disease, mosquitoes can acquire and transmit the virus between infected nonhuman primates (i.e., monkeys) and humans. YF was first discovered in West Africa in 1927 [4]. As of 2023, 34 countries in Africa and 13 countries in Central and South America are either endemic for, or have regions that are endemic for, YF [5]. The World Health Organization (WHO) aims to eliminate the disease by 2026 [6, 7]. The Centers for Disease Control and Prevention (CDC) estimated an annual global burden of approximately 200,000 new cases and 30,000 fatalities, with 90% occurring in Africa [6]. The most common symptoms of YF are fever, hemorrhage, jaundice, vomiting, and failure of the kidney and liver [1]. While only 15% of infected individuals experience clinical symptoms [8], it is estimated that up to 39% of those who experience symptoms die within 7 to 10 days from infection [9, 10].

While the disease was once thought to have a negligible burden on global morbidity and mortality, several recent outbreaks in Sub-Saharan countries have demonstrated its epidemic potential [11, 12]. Between January 2021 and December 2022, 13 African countries (Uganda, Sierra Leone, Republic of the Congo, Nigeria, Chad, Côte d’Ivoire, the Democratic Republic of the Congo, Ghana, Kenya, Niger, and Gabon) reported a total of 203 confirmed and 252 probable cases with 40 deaths (9% case fatality rate) [13]. In general, the number of reported YF cases is expected to be lower than the true disease burden due to the inadequate human capacity and laboratory infrastructure to detect and report cases [14].

**Fig. 1:**
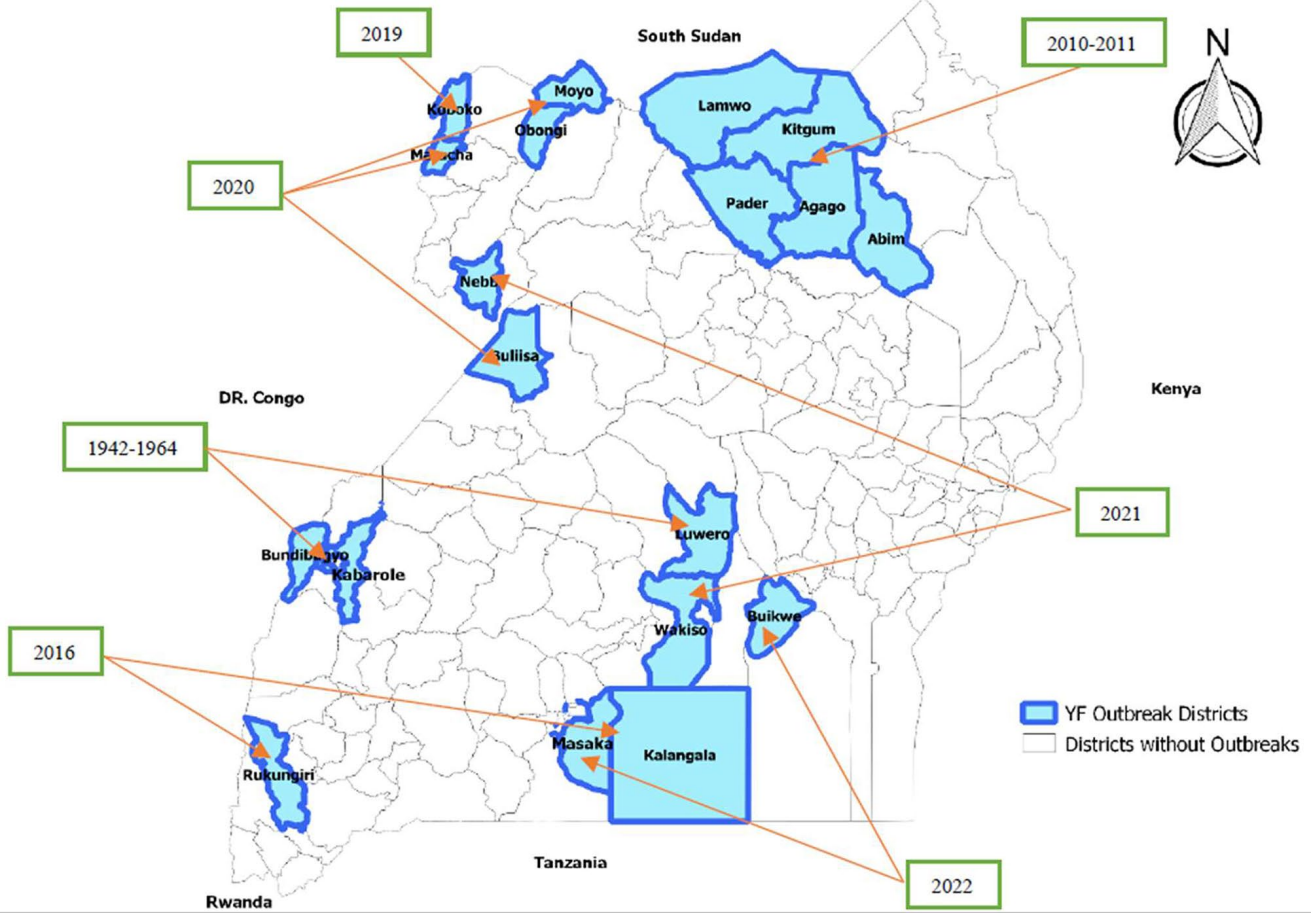
Map of YF outbreaks in Uganda; 1942–2022 [17]

Uganda is located within the African YF belt and is classified among the 27 high-risk countries in Africa for this disease [15]. The first YF outbreak detected in Uganda dates back to 1942 in the Bundibugyo district (Fig. 1) [16]. Uganda’s Ministry of Health has documented at least one YF outbreak per year over the past four years (Table 1). These outbreaks were confirmed in the following districts: Masaka and Koboko in 2019; Moyo, Maracha, and Buliisa in 2020; Nebbi and Wakiso in 2021; and Masaka in 2022 [17]. During the same period, a total of 3,568 suspected cases were reported from health facilities across Uganda. While these outbreaks were partially contained through existing control measures such as targeted mass vaccinations and heightened awareness [17], the ongoing trend of recurring outbreaks is concerning and represents a significant threat to public health and health security in Uganda.

**Table 1:** YF outbreak cases by year and region in Uganda*

Year	Suspected Cases	Laboratory Confirmed Cases	Deaths
1942–1971	4	4	2
2010	181	13	45
2016	42	7	14
2019	2	2	Not Available
2020	Not Available	8	4
2021	Not Available	2	0
2022	Not Available	2	0

*Source: Uganda National Expanded Program on Immunization (UNEPI), Ministry of Health, Kampala, Uganda [17]

One Health is a transdisciplinary approach that recognizes the interconnectedness of human, animal, and environmental health to address complex health challenges collaboratively [18]. Numerous studies have shown that persistent human exposure to the YF virus often a result from economic and occupational activities in and around the forest reserves [16, 19]. This situation is notably observed in Uganda, where individuals are at increased risk of YF due to presence of the animal host reservoir (nonhuman primates) and vector hosts [19], [16]. Moreover, wildlife conservation parks situated near water bodies, such as lakes, are regarded as breeding grounds for mosquitoes that potentially transmit YF virus to susceptible individuals [20]. The persistent recurrence of YF raises concerns about the effectiveness of the current response strategies and approaches implemented by the relevant authorities in Uganda. In this review, we have adopted a One Health approach that delve into the complexity of the issue, evaluate the comprehensiveness of response and preventive strategies, and propose potential interventions across the human, animal and environment domains.

## One Health domains and YF in Uganda

A One Health approach to address complex health issues is increasingly being adopted by health organizations and governments worldwide^,^ [21, 22]. For instance, a One Health approach has been recommended as the basis for infectious disease control programs such as infectious disease control such as Lassa fever in sub-Saharan Africa [23] Uganda has experienced various infectious disease outbreaks, including Ebola, Crimean Congo hemorrhagic fever, Rift Valley Fever, Marburg Virus and YF. Buregyeya et al [24] et al. stated in their article titled “*Operationalizing the One Health Approach in Uganda: Challenges and Opportunities”* that Uganda is considered a ‘hot spot’ for emerging and re-emerging infectious disease [24]. Given the complex interconnections among humans, animals, and the environment, there is a compelling need to implement a One Health approach to address YF [24].

In the animal domain, scientific literature has shown that wildlife, especially nonhuman primates (e.g., monkeys), is the primary reservoir of many emerging and re-emerging infectious diseases including YF [22]. Monkeys play a vital role in harboring and sustaining the YF virus [25]. Consequently, outbreaks and sporadic cases of YF in humans often coincide with epizootics among monkeys [26]. In tropical regions such as in Uganda, the sylvatic (jungle) form of YF prevails as the most predominant variant. Viral transmission is sustained through mosquitoes, which can bite both infected monkeys and humans and transfer the virus to susceptible hosts [27].

In the human domain, human activities and susceptibility due inadequate acquired immunity contribute to the sustained occurrence of YF [28, 29]. For instance, research revealed that changes in land use may influence the shift from sylvatic-to-urban YF transmission cycles [30].

Between 1996 and 2013, 91% of the regions in Uganda, except for the Teso area, experienced a reduction in forest coverage due to agricultural, housing, and industrial activities [31]. This led to disrupting the natural habitats of nonhuman primates and humans living in closer proximity to wildlife including nonhuman primates. Moreover, higher incidence of YF has a consequence of Uganda’s swift economic development which led to rapid urbanization, population growth, encroachment into forests, and climate change [32, 33].

The environmental factors encompass climate and seasonal temperature variation, changes in urbanization and land use, and vector population distribution. Important environmental and bioclimatic data have demonstrated that prevalence of zoonotic diseases is both sustained and exacerbated by shifts in average temperature, total annual precipitation, and relative humidity [30, 34, 35]. The rise in precipitation and warmer temperatures likely expanded mosquito populations in affected regions [30]. Furthermore, the environmental preference of mosquitoes determines the nature of the outbreak. Some mosquitoes breed close to human habitats (domestic breeding), some primarily inhabit jungle (wild breeding), while others thrive in both settings (semidomestic) [27]. Projection model indicated, with 93.0% certainty (95% confidence interval: 92.7–93.2%) that YF mortality in Africa is expected to increase significantly by 2050. due to climate change [36]. Another environmental factor is the malaria mosquito control programs that also impact other breeds of mosquitoes [37]. This suggests that public health measures used to control mosquito-causing malaria (e.g., widespread deployment of insecticide-treated bed nets) could impact the occurrence of YF and vice versa [37, 38] More specially, Uganda is experiencing a steady rise in temperature and variabilities in rain fall pattern. More rainfalls are observed during the dry season. Due to these changes, it is forecasted that the effects of climate change would lead to rise in the incidence and spread of certain mosquito-borne such as YF [39]. Extremely high temperatures have been associated with increase in acute infectious diseases and hospital admissions in certain parts of the country [40]. From a study, environmental risk factors related to YF transmission in Uganda include homes were potentially-water-holding containers, and compounds with standing water. There exist also numerous structural flaws in homes that might encourage mosquito entry, including missing screens in of the ventilators and imperfectly fitted outside doors that could allow mosquitoes to enter [41].

### Limitations of existing control and prevention strategies for YF in Uganda

Uganda’s main approach to control and prevent the disease is mostly through surveillance, case management, and vaccination. YF is monitored through a passive surveillance system that is based on reports from healthcare providers, mostly primary health care clinics [42]. The standard case definitions [43] used for surveillance purposes are provided in Table 2. In the event of an outbreak in Uganda, specific measures are taken, including active surveillance (case finding), case management, and vaccination. Active surveillance includes deployment of human resources to affected areas to proactively search for cases in communities and healthcare facilities, enabling rapid investigation. Case management involves clinical treatment of suspected, probable and confirmed cases in isolated units following standardized operating procedures. Reactive mass vaccination using the YF vaccine is carried out, typically targeting individuals aged 6 months and older in outbreak-affected regions as well as neighboring populations or areas at risk [17, 44]. Table 3 summarize published studies that describe Uganda response to YF outbreaks.

**Table 2:** YF case definitions

Case Category	Standard case definitions
Suspected case	Any person with acute onset of fever, with either a negative laboratory test for malaria or failure to respond to a full course of antimalarials AND one of the following: 1. Jaundice or scleral icterus appearing within 14 days of onset of the first symptoms 2. Bleeding from either the mouth, nose, gums, skin, eyes or stomach (gastrointestinal tract)
Probable case	Any person meeting the suspect case definition with additionally one of the following: • Epidemiological link to a confirmed case or an outbreak • Positive postmortem liver histopathology
Confirmed case	1) Any person who meets the suspect or probable case definition criteria AND has not had YF immunization within 30 days before onset of illness; and one of the following: a) Detection of YF-specific IgM; b) Detection of fourfold increase in yellow-fever IgM, or IgG antibody titers between acute and convalescent serum samples, or both; c) Detection of YF-specific neutralizing antibodies. OR 2) Any person who meets the suspect or probable case definition criteria and has not had YF immunization within 14 days before onset of illness; and one of the following: a) detection of YF virus genome in blood or other organs by PCR b) detection of YF antigen in blood, liver or other organs by immunoassay; c) isolation of yellow-fever virus

**Table 3:** A list of activities reported during outbreaks in 2010 and 2016 to control YF outbreaks

Reference Article	List of activities reported	Year of Outbreak
Epidemiological and laboratory characterization of a YF outbreak in northern Uganda, October 2010–January 2011 [16]	• Case-series investigation (epidemiological and laboratory investigations on suspect cases) • Line-listing and data analysis	2010
YF vaccination coverage following massive emergency immunization campaigns in rural Uganda, May 2011: a community cluster survey [44]	• Mass emergency immunization campaigns in selective districts	2010
Investigation and response to Rift Valley Fever and YF outbreaks in humans in Uganda, 2016 [45]	• Blood sample collection • Referrals • Line listing • Active surveillance	2010
Outbreak of YF in central and southwestern Uganda, February–May 2016 [19]	• Medical records reviewed • Conducted active community case finding. • Case- control study • Entomological studies • Environmental assessments • Reactive vaccination campaign	2016

We here discuss the limitations in Uganda response strategies to YF outbreaks:

1. **Inadequate YF surveillance**: Infectious disease surveillance serves as a crucial epidemiological tool for monitoring disease trends and detecting outbreaks [46]. Uganda has actively adopted and implemented an Integrated Disease Surveillance (IDSR) plan since 2000 [47]. IDSR plan integrates surveillance for priority health issues at all health system levels [43]. Under this plan, diseases, including YF, are expected to be identified within the community and health facilities for thorough investigation and reporting [43]. However, the existing surveillance systems (both YF passive and sentinel surveillance systems) for human population in Uganda face significant challenges such as inadequate laboratory diagnostic capabilities, a shortage of skilled public health professionals such as those needed for data analysis and interpretation of surveillance data, leading to the underreporting of YF cases, and slower outbreak response [48]. Moreover, health workers and community-based surveillance volunteers often lack awareness of the YF case definition, leading to ineffective and irregular reporting of cases at both community and health facility levels [49]. Consequently, only few healthcare units report YF cases [17]. Unlike diseases such as measles and acute flaccid paralysis, active search and reporting of suspected YF cases typically occur during outbreaks, further limiting surveillance efforts. Additionally, the current surveillance focuses exclusively on human populations, neglecting nonhuman hosts and vectors. Moreover, the country’s population growth has led to habitat destruction, fragmentation, and overexploitation of natural ecosystems, resulting in loss of biodiversity and increased risk of zoonotic disease transmission due to closer human-animal interactions and environmental degradation. To address these limitations, developing and implementing a One Health surveillance that integrates existing YF passive and sentinel surveillance systems along with new systems that monitor and report the virus in nonhuman hosts and disease vector is essential. The development and implementation of an integrated One Health surveillance system would not only act as an early warning system, but will improve disease detection in the three health domains (human, animal, and environment) and provide a foundation for informed public health actions.
2. **Reactive mass vaccination campaign**: The YF vaccine, YF-STAMARIL, by Sanofi Pasteur (Lyon, France), has be shown to highly effective with 95% efficacy against the disease in individuals aged 9 months and older [50]. A single dose is generally considered sufficient for lifelong protection. In some cases, a booster dose may be recommended based on specific risk factors or when traveling to high-risk areas. In many areas globally, routine immunization and organized immunization campaigns have been fundamental in controlling and preventing YF. These campaigns, designed to vaccinate a large number of people within a short timeframe to create a herd immunity [51]. However, until the introduction of YF routine childhood immunization in 2022, Uganda’s current approach primarily relies on reactive mass vaccination campaigns initiated in response to outbreaks [51], an approach that does not prevent future disease outbreaks. Due to the focus on reactive measures, herd immunity against the disease remains low, therefore, it does not provide a sustained interruption of the virus’s life cycle leading to a continuous cycle of outbreaks [12, 52]. The needs for a more proactive and comprehensive vaccination strategy to effectively curb YF transmission in Uganda. For example, Ghana has about 88% population immunity against YF. This high-level population is largely attributable to comprehensive vaccination strategies [53] [54].
3. **Geographically localized response**: The geospatial distribution of YF cases (sporadic and outbreak associated) reveals a nationwide burden of this disease in Uganda. Despite this broad geographic impact in the country, responses to outbreaks have typically been confined to the affected districts and nearby areas [17]. For instance, during the outbreak between February and May 2016 in the central and southwestern regions of the country, active vaccination efforts were limited to these affected areas [19]. Given the widespread geographical risk associated with YF, it is imperative to reconsider the scope of outbreak responses. Instead of confining interventions within district boundaries, a more prudent approach would be to extend response efforts beyond these limits. This broader strategy acknowledges the extensive reach of the disease, ensuring a more comprehensive and proactive containment approach that aligns with the nationwide impact of YF in Uganda. In the comprehensive strategy, areas experiencing outbreaks can, however, be prioritize for activities such as vaccination with continuous efforts to immunize the rest of the population based on vaccine availability.
4. **Reactive risk communication**: A notable weak link in Uganda’s effort to control and prevent YF lies in the insufficient communication of disease risk to both health professionals and the larger community. A widespread lack of awareness exists regarding the disease’s case definition and the necessary actions to be taken to initiate case investigations among health professionals [11, 55]. Similarly, community members exhibit a general low-risk perception for infectious diseases, exacerbating the challenges in disease prevention. One of the critical issues is the absence of adequate materials such as fact sheets and posters for health education officers to effectively communicate risks to the general population [56, 57]. Moreover, the priority placed on risk communication is often limited to outbreak periods with a focus on epidemic zones but decreases when there are no active cases [56]. For YF control to be effective in Uganda, a comprehensive and continuous communication strategy must be implemented at all levels of health administration [58]. This strategy should not only address informational needs but also include advocacy efforts aimed at both partners and the general public [59]. Such an all-encompassing communication approach is vital to enhance awareness, improve risk perception, and ensure the sustained engagement of both healthcare professionals and the broader community in YF prevention and control initiatives.
5. **Residential vector control vs. wider vector control**: While *Aedes aegypti*, the mosquito species responsible for transmitting YF virus, is also a vector for other mosquito-borne diseases such as West Nile virus, chikungunya, dengue, and Zika virus [60], current vector control efforts in Uganda predominantly focus on malaria, with emphasis on indoor residual insecticide spraying for mosquito population control [61]. However, YF is primarily spread to humans through the bites of infected *Aedes* and *Hemagogus* mosquitoes found outdoors including in forest areas [5]. Hence, limiting vector density control programs solely to households is inadequate. To effectively curb YF transmission, it is imperative to broaden the scope of vector control efforts. Implementing a combination of indoor measures such as long-lasting insecticide nets and outdoor techniques such as sanitation improvement, mosquito traps, and larvicides can significantly reduce mosquito populations [57, 62, 63]. By adopting a comprehensive approach that targets both indoor and outdoor environments, Uganda can create a more resilient defense against YF, addressing not only households but also the forested and outdoor areas where the disease-carrying mosquitoes thrive through methods such aerial spraying [64].
6. **Knowledge gaps in YF epidemiology**: Disease outbreaks do not occur randomly but follow certain patterns linked to contributing factors [65] A complex interplay of factors involving the host, the agent, and environmental characteristics determine the occurrence of an outbreak [66]. A comprehensive understanding of these factors is pivotal in crafting effective control and prevention strategies. However, critical knowledge gaps persist, particularly concerning the intricate transmission dynamics involving monkeys, mosquitoes, and human host [11]. These knowledge gaps extend to the ability to predict the re-emergence of YF, a disease sensitive to climate conditions, based on meteorological changes such as temperature and rainfall [33] Although environmental and entomological surveys are conducted, they often focus on limited geographic areas, thus offering limited insights [17, 19]. To bridge these gaps and enhance the understanding of YF, it is crucial to expand data collection efforts. This includes gathering information on seroprevalence among humans, conducting entomological viral assessments among vectors, implementing pathogen surveillance among monkeys, assessing the impact of loss of biodiversity and environmental degradation on YF incidence in human and nonhuman species [67]. Similar integrated activities are currently used in Tunisia to control arboviruses [68]. A comprehensive approach integrating these data sets could significantly contribute to understanding YF epidemiology and the development of accurate risk assessment models.

## Future directions – proposed strategies within the One Health framework

To address the limitations in Uganda’s current YF control and prevention efforts, we propose the following One Health approach strategies:

1. **Comprehensive surveillance and enhanced laboratory capacity for YF control**: To effectively control YF, Uganda ought to establish a robust diagnostic and disease surveillance mechanisms among both humans and monkeys [69]. Strengthening the existing human population surveillance is paramount through health worker sensitization to case identification, notification, investigation, and prompt reporting. Additionally, laboratory training should emphasize specimen collection, processing, storage, and transportation (including cold chain logistics) to designated reference laboratories. Expansion surveillance activities to include wildlife is a vital aspect of a comprehensive One Health approach to manage re-emergence of YF [70, 71]. For animal surveillance, wildlife staff, including veterinarians, should receive training to identify YF symptoms among monkey populations for early recognition and reporting. This can enhance the prevention of potential spread of the virus to the human population [70, 72]. By integrating enhanced surveillance techniques, fostering collaboration between human and wildlife sectors, and bolstering laboratory capacities, Uganda can significantly strengthen its efforts to monitor, prevent, and control YF outbreaks effectively.
2. **Accelerating routine vaccination uptake campaigns**: The YF vaccine offers a highly effective means of protection, offering a full immunity 30 days postvaccination [73]. The vaccine is considered safe and inexpensive. A single dose is sufficient to offer lifetime immunity [74]. In a positive recent development, Uganda has introduced YF vaccination in its routine immunization schedule, targeting children at 9 months of age [75]. However, the children and adults that have not been vaccinated or previously infected remain susceptible.

Proactive collaboration between the Ministry of Health and relevant sectors is essential to execute comprehensive nationwide immunization campaigns. This strategic move is vital because increasing population (i.e., herd) immunity through mass vaccination and sustaining it with routine vaccinations can reduce the occurrence of outbreaks [74, 76].Furthermore, prioritizing regular vaccination efforts for high-risk populations, such as health workers, wildlife/forestry workers, and environmental health officers, is imperative. With a sustainable nationwide immunization program, Uganda can effectively mitigate the risk of YF transmission and establish a strong defense against potential outbreaks.

3. **Integrated Mosquito Management for YF Control**: Implementing vector control measures targeted at mosquitoes is one of the effective strategies to halt the spread of YF [77]. Domestic YF-spreading mosquito species often breed in man-made environments such as cans, bottles, tires, and clogged gutters. Additionally, epidemiological studies underlined the significance of outdoor infection sources, primarily through daytime mosquito bites [78]. The primary strategies employed to control mosquito populations include indoor spraying of insecticides to reduce adult mosquito populations and applying larvicides to outdoor stagnant water and other potential breeding sites to kill eggs [79]. Beyond these strategies, Uganda can enhance its efforts through the adoption of the Integrated Mosquito Management (IMM) strategy [80]. The IMM strategy encompasses various measures such as public education, vector surveillance, mosquito source reduction, chemical control, and biological modification [81]. Public education empowers communities to combat mosquito breeding in their surroundings. Vector surveillance identifies mosquito species, their populations and locations, guiding appropriate interventions. Skilled workers actively seek and eliminate mosquito larval habitats in source reduction efforts. Chemical control involves safe pesticide usage, ensuring ecological safety [80]. Biological methods, including soundwave instruments and mosquito-eating fish species such as *Gambusia affinis* and *Pimephales promelas*, offer eco-friendly solutions, as demonstrated previously for successful control of another mosquito-borne disease (i.e., West Nile virus) [81]. By integrating these strategies, Uganda can effectively control mosquito populations, minimizing the risk of YF virus transmission and creating a robust defense against the disease.
4. **Heightened continual risk communication**: Effective and constant risk communication is pivotal in addressing the heightened risk of YF. Risk communication involves timely dissemination of knowledge, advice, and perspectives to healthcare professionals and individuals at risk of the disease [82]. Given the nationwide threat posed by YF, it is imperative to inform the general population, especially those residing in forest areas. The Ministry of Health in Uganda can take the initiative to develop and distribute informative materials, including job aids, fact sheets, and posters for public education. Health professionals need training and empowerment to incorporate YF-related topics into routine health education activities at outpatient clinics and during community health outreach programs. Moreover, individuals working in wildlife conservation areas should be educated about the importance of wearing protective clothing such as long sleeves, long trousers, and socks at all times [83]. This comprehensive approach to risk communication plays a vital role in promoting awareness, prevention, and protection against YF. By ensuring that accurate information reaches both healthcare professionals and the general population, Uganda can empower its citizens to make informed decisions, adopt preventive measures, and actively participate in the collective effort to combat YF effectively.
5. **Determination of YF risk factors in human and nonhuman populations**: A comprehensive understanding of disease transmission dynamics is crucial for tailoring effective public health strategies [32]. This understanding encompasses a deeper knowledge of the ecological elements influencing transmission dynamics, factors driving re-emergence, severity, and the adaptation of YF virus. Employing methods such as risk analysis, entomological surveys, seroprevalence studies, and statistical modeling of available data is essential [84]. Environmental factors such as rainfall patterns, vegetation, temperature, presence of mosquito vectors, and susceptible monkey species are critical considerations in developing intervention strategies to reduce the disease burden [85]. Entomological risk assessment must quantify indicators related to the nationwide abundance and geographic distribution of the YF-mosquito population [86]. Seroprevalence studies are invaluable in gauging population exposure, immunity, and susceptibility levels [87]. Statistical modeling techniques can aid in identifying YF risk factors in the country, providing crucial insights for targeted interventions. By employing these multifaceted methods and considering a broad spectrum of factors, Uganda can develop nuanced and precise strategies to mitigate YF risks in both human and nonhuman populations. This approach is fundamental for proactive disease management and prevention efforts. YF risk factors can change overtime or exacerbates due to the impact of climate change and associated drivers linked to anthropogenic forces Therefore, integrated surveillance system is required to monitor such changes.

## One Health stakeholders and their potential contributions to YF control and prevention

To effectively combat YF in Uganda, collaboration and communication among various stakeholders is imperative. One Health approach will ensure that stakeholders go beyond their usual boundaries and work together to understand the systemic drivers of the issue and develop collaborative strategies for the disease control and prevention [12, 22]. We propose establishing a One Health YF Elimination Taskforce, under the leadership of the Ministry of Health. The YF Elimination Taskforce would adopt the National Ebola Taskforce model [88]. Funding for its operations would be spearheaded government and through supports of partners as in the model being adopted [88]. In line with the global strategy to eliminate YF epidemics (EYE) [59], this taskforce would oversee coordination of existing and new YF elimination initiatives, including developing and implementing a comprehensive surveillance system, planning and coordination of YF vaccination campaigns, assessing and integrating mosquito management strategies, enhancing constant risk communication, and determining YF risk factors. The establishment of such a taskforce will require collaboration and coordination among relevant stakeholders in Uganda. Creating an autonomous, high-level expert committee comprising representatives from diverse sectors and disciplines can offer guidance on best practices and their practical implementation. This includes devising effective frameworks to enhance current surveillance systems, aligning them, setting targets, refining monitoring and evaluation processes, and offering evidence-based support during outbreak responses. Additionally, the committee can assess the cost implications and feasibility of these systems. We identified 13 key stakeholders and provided information on their current core mandates and proposed roles in YF control and prevention as follow:

1. **Ministry of Health (MoH)**: The Ministry of Health is responsible for policy review and development, healthcare supervision, resource mobilization, and providing guidance on health issues to other government and non-government agencies. The Ministry’s role would be to lead the YF Elimination Taskforce, mobilizes partners, and coordinates subnational health units.
2. **Health Information Systems Program-MoH**: This program aims to adopt long-term and comprehensive information systems for public health. The program roles in YF control will be developing an integrated alert management system for animal and human surveillance, implement electronic surveillance, and monitor reporting rates.
3. **Uganda Wildlife Authority**: This authority is responsible for wildlife management and preservation within and outside protected zones. The authority role will include overseeing viral surveillance activities among wildlife animals in national parks as part of YF control efforts including laboratory facility for diagnosis of zoonotic diseases, especially in wildlife [89].
4. **Uganda National Expanded Program on Immunization-MoH**: This program’s aim to ensure appropriate immunization for infants and high-risk populations against vaccine-preventable diseases. The program’s role (as part of this existing role) would be operational planning and budgeting to ensure a constant supply of the YF vaccine for achieving routine and mass immunizations, developing immunization guidelines for health workers, and raising awareness among health professionals about YF surveillance [17].
5. **Public Health Emergency Operations Center**: This center was established by the Ministry of Health to coordinate planning and responding to health emergencies. The Center’s roles in YF control will include effective coordination of disease-related emergencies, training district surveillance teams on outbreak management, and enhancing capacity.
6. **Uganda Field Epidemiology Training Program - UFETP**: This program delivers effective and sustainable training programs in field epidemiology. The program’s role in YF control will includes training health professionals on surveillance, providing outbreak support, and offering funding support toward human capacity building/training.
7. **National Environment Management Authority of Uganda**: This authority develops environmental policies, legislation, and recommendations. This authority role in YF control will involves enacting environmental policies to aid in the elimination of mosquito breeding sites.
8. **Uganda Virus Research Institute - UVRI**: UVRI conducts and supports studies in collaboration with academic institutions on human infectious diseases caused by viruses and offers professional guidance. The role of UVRI in YF control (based on their existing activities) will includes designing and implementing national entomological assessments and seroprevalence studies, serving as a national reference laboratory, developing training manuals for health professionals on specimen collection, storage, and transportation to the reference laboratory [90].
9. **National Forestry Authority**: The National Forestry Authority is responsible for the protection and conservation of forest reserves in Uganda. This authority’s roles in YF control will include regulating economic and occupational activities in and around forest reserves, supporting pathogen surveillance (regular sampling of NHP to determine the presence and obtain genomic data of the YF pathogen) in forest zones and limiting interactions between monkeys in forests and nearby communities.
10. **Vector Control Division-MoH**: The division contributes to eradication of malaria through vector management and performs other pest management crucial for public health, particularly in metropolitan areas. In YF control, the organization will help design outdoor vector control strategies.
11. **United Nations Children’s Fund (UNICEF) - Uganda Country Office**: UNICEF advocates for children’s rights and supports their essential needs. UNICEF’s role in YF control in Uganda will includes providing technical support for risk communication activities, supporting the promotion of YF routine immunization through funding, and assisting in mass media advertisements.
12. **World Health Organization - Uganda Country Office**: WHO collaborates with governments, partners, and individuals to advance health and ensure global security. WHO Uganda country office role in YF control will includes offering technical guidelines for disease control strategies, providing funding support for routine surveillance activities, and promoting mosquito net ownership and usage.
13. **Centers for Disease Control and Prevention - Uganda**: CDC strengthens Uganda’s capacity to prevent, diagnose, and respond to public health threats through collaboration. The CDC’s role in YF control will includes providing technical support/expertise for outbreak response and offering funding.

Efforts were made to closely match propose roles with exist mandate or function of organizations. However, the taskforce could support additional training if necessary or even re-assign roles among partner. Through collaboration and the collective efforts among the listed stakeholders, Uganda can implement a comprehensive, multidisciplinary One Health approach to YF control, ensuring a more effective response to the disease threat.

## Conclusions

The review underscores the persistent threat of YF in Uganda, marked by frequent outbreaks that challenge the nation’s health security. Identified deficiencies in surveillance, vector control, and population immunity necessitate a transformative multi-sectoral approach. In light of this, we advocate for the adoption of a One Health approach, emphasizing collaboration and communication among various diverse disciplines and stakeholders in human health, animal health, and environmental health. This approach is pivotal in crafting comprehensive and proactive disease control strategies, transitioning from localized reactive responses to a harmonized, coordinated approach. By implementing the proposed strategies and fostering coordination and collaboration among stakeholders, we anticipate significant positive outcomes. These include fewer outbreaks, enhanced surveillance, proactive vaccination campaigns, elevated population immunity, reduced viral transmission in wildlife, decreased mosquito populations, a more skilled public health workforce, and improved disease control infrastructure. Overall, the adoption of the One Health approach is expected to substantially reduce the morbidity and mortality associated with YF, fortifying Uganda’s resilience against this persistent health threat and form as a base for controlling other infectious diseases.

## Data Availability

Not applicable.

## Abbreviations

YF: YF
WHO: World Health Organization
CDC: Centers for Disease Control and Prevention
IDSR: Integrated Disease Surveillance
IMM: Integrated Mosquito Management
MoH: Ministry of Health
UFETP: Uganda Field Epidemiology Training Program
UVRI: Uganda Virus Research Institute
UNICEF: United Nations Children’s Fund
